# Lectures on the Exploration and Treatment of Diseases of the Chest

**Published:** 1840-07-11

**Authors:** W. W. Gerhard


### MEDICAL EXAMINER.

DEVOTED TO MEDICINE, SURGERY, AND THE COLLATERAL SCIENCES.

No. 28.] PHILADELPHIA, SATURDAY, JULY 11, 1840. [Vol. III.

### LECTURES ON THE EXPLORATION AND TREATMENT OF DISEASES OF THE CHEST.

BY W. W. GERHARD, M. D.

### Lecture IX.—*Bronchitis.*

Having concluded the subject of pleurisy in its various forms, I might now take up that of pneumothorax, as in this affection the same membranes are involved; but inasmuch as bronchitis is of more frequent occurrence, and, like pleurisy, in very many cases complicates or gives rise to affections of the parenchyma of the lungs, I think it comes in very well in this place, and I shall therefore now proceed to treat of this disease. The term *bronchitis* is, in common parlance, applied to various affections of the respiratory organs, as laryngitis, several affections of the lungs, &c.; but it should never be used in this way by physicians, as it is vague and unphilosophical: the term should be confined to inflammation of the mucous membrane of the bronchial tubes.

Bronchitis, like all other inflammations, is divided into *acute* and *chronic.* The acute has been subdivided in reference to the greater or less quantity of the secretion, and its epidemic or sporadic nature. The first division is of very little importance; but the second is well founded, as the disease is much more serious when it occurs in an epidemic form. In the epidemic bronchitis, to which the name influenza has been given, the severity of the constitutional symptoms is by no means proportioned to the intensity of the local lesion,—the latter in many cases being very slight, while the former are sufficient to confine the patient to his bed for several days. The constitutional symptoms are pains in the back, &c., high fever, and extreme prostration.

We have no opportunities of examining the anatomical lesions in simple acute bronchitis, as the disease is seldom or never fatal. On this account we can only study them in cases in which it is secondary to other grave diseases, and in these cases we often meet with every stage of bronchitis. In this disease the mucous membrane itself is chiefly involved, and not the subjacent tissue, as is the case in serous inflammations. The lesions observed are injection of the mucous membrane, ecchymoses, thickening, and induration. The last mentioned lesion is inferred from analogy to occur in primary acute bronchitis, as it cannot be demonstrated; but in cases where the affection is secondary to some other disease, we frequently meet with it. These lesions are more marked in the minute than in the large bronchial tubes, although the signs of inflammation of the larger tubes may have been very decided before death.

In anemic patients, the mucous membrane, instead of presenting increased redness, is found to be pale; the only change which is perceived is the opacity of the membrane, which, in a healthy state, is almost transparent. This appearance is not at all uncommon in persons whose blood is deficient in red globules at the time of the occurrence of the affection.

In acute bronchitis ulceration rarely takes place, although it is by no means infrequent in the chronic form of the disease. It is almost entirely confined to those acute forms which have a specific character, such as bronchitis, complicating rubeola and variola. In these cases the ulcers are at first confined to the follicles, although they sometimes extend themselves, and acquire an irregular outline, involving the surrounding membranes. Ulceration affects principally the trachea and larger tubes, where the follicles are well developed, and rarely extends to the minuter ramifications of the bronchial tree. I shall not dwell upon this lesion at present, as it does not deserve much attention in this place. There is, however, another modification of much more importance, viz. the effusion of lymph and formation of false membrane. This form of inflammation, which has been termed diptheritis, occurs also in severe cases of croup.

When bronchitis commences in the small tubes, and extends upwards towards the larynx, it is not unfrequently fatal; but when it follows the opposite course, beginning at the larynx, and extending downwards, it may be arrested, and the disease will almost always terminate favourrably. Inflammation of the bronchial mucous membrane is in some cases attended with a serous effusion, which, occurring under the membrane, gives rise to œdema: when this takes place in the upper portion of the larynx, it constitutes œdema of the glottis. Bronchitis tends, in most cases, to get well without the formation of pus. Its progress is as follows: at the commencement of the inflammation the membrane is injected and thickened, and its secretion is arrested. An increased secretion then takes place, which is intended by nature to relieve the turgescence of the vessels: if the inflammation continues, the secretion then becomes opaque; if it is not arrested at this stage, but still goes on, purulent globules are mixed with the mucus, and in more protracted cases pure pus is secreted. The expectoration, however, is never found to consist of pus alone, because, although certain parts of the membrane secrete pure pus, yet before it is expectorated, it is mixed with mucus from other portions.

Bronchitis may occur as a primary disease, or as secondary to some other affection of the lungs. When it occurs as a primary affection, it may either terminate in perfect recovery, or may give rise to the development of some lesion of the parenchyma of the lungs, such as pneumonia or phthisis. The former is the more common termination, but the latter is not unfrequent.

In other cases the bronchitis supervenes on one of these affections. This distinction is of the greatest importance in forming a prognosis; for when the disease is secondary, it is merely a part of the tuberculous disease; when it precedes this affection, it may proceed to a certain length, and the tubercles may then be arrested.

We now come to the signs which indicate acute bronchitis. These may be divided into *general* and *local.* The general signs are febrile excitement, with its attendant symptoms of enfeebled strength. The local signs are cough, expectoration, soreness of the chest, with the physical changes in the respiratory sound. In treating of the local signs, I shall first consider those connected with obstructions to the passage of air through the tubes. The sonorous rhonchus is generally heard in the first stage of acute bronchitis: it is produced by the thickening of the mucous membrane of the larger portions of the tubes, which contracts their calibre, and thus impedes the passage of air through them. I described this rhonchus in a previous lecture, and pointed out its distinctive characters. As it often occurs first at the root of the lungs, where bronchial respiration is loudest in pneumonia, you may, without you are attentive to the distinctive characters which I laid down, mistake it for the latter sound: it is important to bear this in mind. The sonorous rhonchus is heard in the larger tubes; but when the inflammation extends to the smaller tubes, a sibilant rhonchus is produced, which is caused by the same physical condition as the sonorous, but differs from it on account of the smaller calibre of the tubes in which it occurs.

Although these rhonchi are very frequent, yet if you expect to meet with them in all cases of acute bronchitis, you will be egregiously mistaken, because the thickening must reach a certain point before the sound is developed, and therefore if it does not proceed thus far, no rhonchus will be heard. Feebleness of respiration is a more constant sign in bronchitis: it results from the air not passing freely through the tubes; and, like the rhonchi themselves, this sign is extremely variable, shifting from one portion of the lung to another, as it is temporarily influenced by the efforts of breathing, which force the air into the lungs, and for a time clear the tubes. In this affection, the chest sounds perfectly clear on percussion in the first stage; it, however, becomes somewhat dull in the second, but the alteration is very slight.

In the second stage of the disease, secretion takes place into the bronchial tubes, which gives rise to the moist rhonchi, mucous and subcrepitant. The former, like the sonorous rhonchus, is produced in the larger bronchial tubes,—the latter in the smaller. The subcrepitant rhonchus resembles very much the crepitant, which is peculiar to pneumonia: this renders the diagnosis somewhat difficult, as the cases in which it occurs, simulate pneumonia very much. When, however, the bronchitis is of considerable extent, it does not resemble pneumonia so closely, for the latter disease scarcely ever extends to a large portion of both lungs, as is often the case with bronchitis.

After the secretion from the mucous membrane occurs, the thickening subsides, and the respiration gradually returns to the normal state, but, for a time, it may be more or less mixed with moist rhonchi,—that is, the mucous and subcrepitant. These gradually cease as the resolution of the disease advances.

The expectoration in acute bronchitis is very variable: at first, as the cough is dry, there is little or no expectoration; but as the disease advances towards resolution, or passes into a more chronic variety, the expectoration becomes much more abundant, and consists of sputa which are almost peculiar to this disease. When the disease is still slight, or if it remain stationary, the sputa are generally transparent, and consist merely of thin mucus. As soon as it tends decidedly towards resolution, or if, instead of tending towards resolution, it assumes a sub-acute form, and becomes chronic, the character of the sputa changes,—they become more thick and opaque, and of a whitish colour. If the disease be very intense, a small quantity of purulent matter is sometimes mixed with the sputa, and they assume the muco-purulent character. In these cases their form is irregular, and the thicker portion is generally diffused in irregular shreds through the thinner part. As the disease declines, the sputa gradually become less and less abundant. If the inflammation be very violent, the secretion from the bronchial tubes becomes almost of the consistence of coagulable lymph, and is firm, and moulded into the form of the bronchial tubes; these tubes, or polypi, as they are sometimes called, indicate a high degree of inflammatory action.

The local signs of primary acute bronchitis, differ but little from those of other forms of the disease, such as the chronic, &c., but the general signs are somewhat different,—they are generally very well developed in epidemic cases, and are very slight in the sporadic. The patient is first taken with a chill, which is followed by febrile excitement, thus resembling other inflammations, as well as those of serous membranes and the substance of the lungs, although it is of much less intensity. The patient, then, has slight fever, and sensations of chilliness occuring at different times, restlessness, heat in the palms of the hands, &c. The condition of the pulse is in perfect correspondence with the moderate fever, rarely exceeding eighty or ninety in the minute. In epidemic bronchitis the condition of the patient may be very different; the pulse is often small, compressible, and frequent; there is great prostration and disturbance of the nervous system; and, consequently, the tolerance of loss of blood is much less than in serous inflammations. There are other symptoms depending upon the febrile excitement, such as anorexia, thirst, and headach.

There is another set of symptoms which is secondary, and belongs to affections of the other tissues, principally the serous: of these the inflammation of the pleura is the most common, producing pain, which is increased during the act of inspiration. The pleurisy supervening on or complicating bronchitis, is very slight, and is usually dry; when the pleurisy is considerable, it is looked upon as the primary disease, of which the bronchitis is a complication. This accidental pleurisy may prove a cause of death in certain cases; when, for instance, there is hypertrophy of the heart, or when the patient is loaded with fat, it produces this catastrophe by increasing the dyspnœa which usually attends bronchitis, when it attacks the same individuals. The danger in these cases arises chiefly from the pain which impedes the respiration: in simple bronchitis the pain is slight, and often limited to a mere soreness.

Acute bronchitis generally lasts but a few days, and its termination is in most cases favourable. It sometimes, however, runs into the chronic form. This may depend upon the peculiar susceptibility of the patient to inflammation of the mucous membrane, or the unfavourable hygienic circumstances in which he is placed. In some cases it leads to the development of tubercles in the lungs; this, most commonly, is owing to a decided tuberculous diathesis of the individual affected with it.

The treatment of bronchitis is simple, and will occupy us but a short time. You will find in books generally a regular course laid down for the treatment of this affection,—the first step of which is, in severe cases, the abstraction of blood. Bleeding is unquestionably a most useful remedy; but it should not be prescribed for all patients indiscriminately, for the milder cases get well very rapidly without it. We should only resort to it in severe cases, for there are other means by the use of which we may cause the disease to abort. These consist chiefly of the nauseating and stimulant expectorants and diaphoretics. In most cases I prefer the vegetable diaphoretics, aided by hot pediluvia, and generally make use of an infusion of eupatorium and sanguinaria, or eupatorium and seneca, after the following formula:

℞. Eupator. Perfol. } āā ℥ss.

Rad. Senegæ }

M., et infunde in aq. bull Oj.

A tablespoonful or two may be given every nour, or a larger dose less often. Ipecacuanha and tartarized antimony produce a decided effect on the disease. The latter is not always well borne, and ought to be used in large doses only in severe cases, as it may cause much irritation of the stomach. I give it usually in very small doses, sometimes in lemonade or neutral mixture, the object not being to excite severe nausea, but to produce a sedative effect. Dr. Physick has the credit of originating a remedy which was much used at the Almshouse Hospital some years ago. It consists of tartarized antimony gr. ij., bitartrate of potassa ℥ij., dissolved in one quart of flaxseed tea, to be taken in divided doses, in the course of twenty-four hours. This remedy is not altogether safe; for if the patient should drink a large quantity of it through mistake, it would probably produce very unpleasant symptoms, as tartarized antimony diffused in a large quantity of any fluid is very apt to bring on violent inflammation of the mucous membrane of the alimentary canal, though the quantity taken be not very large. It may be advantageously combined with opium. Some give a dose of opium alone in the commencement of the affection;—I prefer, however, this combination, which produces diaphoresis, and often affords very speedy relief. You may give a fourth of a grain of tartarized antimony, with one-seventh of a grain of sulphate of morphia, or you may vary this to suit the case.

When the disease does not subside at once, after active treatment, the patients generally ask for something for their cough. In these cases many cough mixtures are used, most of which are beneficial in their effects. They contain a narcotic, nauseating, or stimulating ingredient, and sometimes a combination of these, commonly mixed with mucilage of gum arabic, which fulfils the indication of allaying the irritation about the throat. A remedy in very general use is the *Brown mixture*, the composition of which you are well acquainted with. Another common mixture is one, of the syrups of seneca and squills, to which opium may be added if necessary; but you should be very cautious about giving opium in mixtures to children, as the accumulated effect of repeated doses may arrest the secretions, and produce other dangerous results. Certain stimulants are frequently given with advantage towards the close of acute cases, and are very useful in the chronic forms of the disease; these are gum ammoniac, balsam of Tolu, balsam of copaiba, &c. The precautions necessary to be observed in convalescence, are the same as in other acute diseases. The general indications, therefore, in the treatment of bronchitis, are, if possible, to bring about a cure of the disease by resolution; this rarely takes place without a secretion of mucus from the membrane. Hence, if you prevent the fever and local inflammation from running sufficiently high to impede secretion, either by bloodletting, or nauseating, or stimulating diaphoretics, you produce nearly the same effect. After this object is attained, the local stimulants which tend towards the lungs favour very much the secretion of mucus, which is almost essential for the removal of the disease.

There are several circumstances which modify this affection to a considerable degree. The most important of these is age,—the bronchitis of children and of old men being very different from this disease as it occurs in adults.

The bronchitis of children is particularly interesting; it extends usually from the trachea down to the tissue proper of the lungs, involving the whole mucous membrane of the large and small bronchial tubes. Its chief peculiarity is its tendency to pass into lobular pneumonia; indeed, if the bronchitis continue for a considerable length of time, this affection is almost certain to supervene. Secretion takes place very early, and consequently the dry rhonchi do not make their appearance, or continue for so short a time that they escape observation: this is another point in which it differs from the affection as it occurs in adults. As the smaller bronchial tubes are usually affected, we almost always find the subcrepitant rhonchus, which can be heard at all times, for children do not expectorate, but throw off the accumulated secretion by an effort of vomiting. The chest usually gives a clear sound on percussion, though it is sometimes rendered dull by the accumulation of mucus in the small tubes, and of blood in the tissue of the lungs. These signs are more marked, and more early developed in the right lung, which is more commonly the seat of pneumonia than the left. Besides the physical signs, we meet with a loose cough, orthopnœa, and flushing of the face, which, instead of being circumscribed as in the case of adults, extends over the whole face, and is of a purplish colour, which is to be ascribed to the imperfect aeration of the blood. There is also at times great febrile excitement, with cerebral symptoms.

